# Intranuclear inclusions in a fragile X mosaic male

**DOI:** 10.1186/2047-9158-2-10

**Published:** 2013-05-21

**Authors:** Dalyir I Pretto, Michael R Hunsaker, Christopher L Cunningham, Claudia M Greco, Randi J Hagerman, Stephen C Noctor, Deborah A Hall, Paul J Hagerman, Flora Tassone

**Affiliations:** 1 Department of Biochemistry and Molecular Medicine, School of Medicine, University of California at Davis, One Shields Avenue, Davis, CA, USA; 2 Department of Psychiatry and Behavioral Sciences, School of Medicine, University of California at Davis, Davis, CA, USA; 3 MIND Institute, University of California Davis Medical Center, Sacramento, CA, USA; 4 Neuroscience Graduate Program, University of California, Davis, Davis, CA, USA; 5 Department of Pathology, School of Medicine, University of California at Davis, Davis, CA, USA; 6 Department of Pediatrics, School of Medicine, University of California at Davis, Davis, CA, USA; 7 Department of Biochemistry, Neurological Sciences, Rush University, Chicago, IL, USA

**Keywords:** Intranuclear inclusions, FXS, FXTAS, Premutation

## Abstract

Lack of the fragile X mental retardation protein leads to Fragile X syndrome (FXS) while increased levels of *FMR1* mRNA, as those observed in premutation carriers can lead to Fragile X- associated tremor ataxia syndrome (FXTAS). Until recently, FXTAS had been observed only in carriers of an *FMR1* premutation (55–200 CGG repeats); however the disorder has now been described in individuals carriers of an intermediate allele (45–54 CGG repeats) as well as in a subject with a full mutation with mosaicism.

Here, we report on molecular and clinical data of a male *FMR1* mosaic individual with full and premutation alleles. Molecular analysis of *FMR1* and FMRP expression in this subject is consistent with a FXS phenotype. We observed reduced expression of FMRP in both peripheral blood and brain leading to the FXS diagnosis. In addition, a dramatic 90% depletion of both *FMR1* mRNA and FMRP levels was observed in the blood, as normally observed in FXS cases, and an even greater depletion in the brain. A clinical report of this patient, at age 71, described neurodegenerative signs of parkinsonism that were likely, in retrospect, part of a FXTAS scenario as post-mortem examination shows the presence of intranuclear inclusions, the hallmark pathology of FXTAS.

The findings presented in this study indicate co-morbidity for both FXS and FXTAS in this individual carrying both full and premutation *FMR1* alleles. In addition, based on symptoms and pathological and molecular evidence, this report suggests the need to redefine the diagnostic criteria of FXTAS.

## Introduction

Two very different disorders arise from a CGG repeat expansion mutation at the promoter region of the X-linked *FMR1* gene: Fragile X syndrome (FXS) and Fragile X -associated tremor ataxia syndrome (FXTAS).

Full mutation (FM) individuals with greater than 200 CGG repeats invariably develop FXS, a neurodevelopmental disorder that is present from birth and produces cognitive impairment, behavioral, emotional and sleeping problems [1-3]. Additionally, approximately 60% of children with FXS can develop autism spectrum disorders (ASD) [4,5]. This expansion mutation usually causes total methylation of the *FMR1* gene, which consequently becomes silenced, leading to the absence of the *FMR1* protein (FMRP), the underlying cause of FXS.

Individuals with shorter ‘premutation’ (PM) expansions in the *FMR1* gene, ranging from 55–200 CGG repeats, usually do not have developmental disabilities but are at high risk for developing FXTAS in late adulthood [6]. FXTAS is a late-onset neurological syndrome affecting older males and females over 50 years of age and presenting features such as action tremor and ataxia, cognitive decline, neuropathy, autonomic dysfunction and parkinsonism [7]. The neuropathological signs of FXTAS include white matter disease and Purkinje cell loss in the cerebellum. Further, the presence of eosinophilic intranuclear inclusions throughout the brain [8,9], in testis [10] and in other organs has been reported in both humans [11] and in the CGG KI mouse model of PM [12].

PM alleles are associated with increased transcription of the *FMR1* gene and toxic accumulation of CGG-repeat expanded mRNA that is thought to contribute to the formation of intranuclear inclusions and to the pathogenesis of PM-associated disorders, particularly FXTAS. The exact mechanism of mRNA-mediated neurotoxicity remains incompletely understood. One possibility is that CGG binding proteins are sequestered in the intranuclear inclusions, which also contain *FMR1* mRNA [13]. More than 30 such sequestered proteins have been identified within the intranuclear inclusions [14-16]. Included are Sam68 and the DROSHA/DGCR8 complex which play a key role in the biogenesis of miRNA and which expression pattern has been found altered in individuals with FXTAS [16,17]. However, the sequestration hypothesis may not fully account for the pathogenesis of FXTAS. PM carriers can also exhibit reduced FMRP levels, particularly in the upper PM range [18-21], which can lead to FXS features.

Since the first FXTAS cases were described [22] it was thought that the syndrome was exclusively limited to PM carriers. However, very recent studies reported FXTAS in carriers of intermediate alleles (45–54 CGG repeats) [23,24] and in a male with methylation mosaicism [25]. Thus, since FXTAS has been linked to toxicity led by elevated *FMR1* mRNA an association, although less striking, between transcriptionally active *FMR1* expanded alleles across the whole CGG repeat range and FXTAS could be made. Indeed, cases of individuals who meet diagnostic criteria of FXTAS but not falling within the “PM category” have been reported. These constitute a group of individuals in whom neurological manifestations seen in the PM related FXTAS spectrum exist. Another question concerns the presence of intranuclear inclusions in carriers of alleles outside the premutation range. In fact, rare and small intranuclear inclusions were observed in three males with FXS [26]. Intranuclear inclusions typically occur in FXTAS and are considered one of the major diagnostic criteria of FXTAS [27], but the presence of intranuclear inclusions in carriers of alleles outside the premutation range including intermediate and FM alleles demonstrates the need to redefine the diagnostic criteria of FXTAS so that these alleles are included.

Here we report molecular, neuropathological and clinical characterization of a man with *FMR1* size mosaicism. *FMR1* size mosaicism indicates that a person carries both FM and PM alleles. The subject developed FXS and showed signs of neurodegeneration during aging. A previous clinical study on this case reported that the patient developed parkinsonism that was not thought to be attributable to FXTAS [23], although parkinsonism is often co-morbid with FXTAS. This patient also experienced progressive cognitive decline, which is common in FXTAS. Molecular post-mortem examination determined the presence of PM and FM alleles in both peripheral blood and brain, although in different proportions of cells. Importantly, we observed intranuclear inclusions, the hallmark of neuropathology in FXTAS, in the cerebellum, frontal cortex and hippocampus of this individual.

Our molecular and neuropathological observations and re-assessment of clinical reports suggest that this individual was affected with both FXS and FXTAS.

## Methods

### Molecular measures

#### Consent

Written informed consent for publication of his case report and any accompanying images was obtained from the patient earlier in life or from a family member. Tissue harvest and brain autopsy was performed in accordance with University of California, Davis, Institutional Review Board-approved protocols.

#### CGG repeat sizing

CGG repeat size was measured on genomic DNA extracted from peripheral blood and from brain regions using standard procedures (Qiagen, Hilden, Germany) and determined by Southern Blot and PCR analysis as previously described [28,29].

#### FMR1 mRNA expression levels

Total RNA from all brain regions was isolated using trizol (Life Technologies, Carlsbad, CA). cDNA synthesis and QRT-PCR used to quantify *FMR1* mRNA levels were as in [30].

#### FMRP expression

##### Western blot analysis

Postmortem cerebellum, frontal pole, orbital frontal cortex, medial frontal cortex and dorsolateral pre-frontal cortex tissues from this case and from the cerebellum of 8 age matched controls (CGG ranging from 21 to 48 repeats) were pulverized in liquid nitrogen and resuspended in 1X RIPA buffer (Cell Signaling, Beverly, MS) that contained protease and phosphatase inhibitors. The lysates were sonicated and spun in a Sorval centrifuge at 18,000 rpm, 4°C. The supernatants were collected and protein concentrations measured using a BCA protein assay (Thermo Scientific, Pierce Biotechnology, Rockford, IL). Approximately 65 μg of protein were loaded in a 10% Criterion SDS/PAGE gel and ran in 1X MOPS at 25 mAmps for 30 minutes followed by 80 volts for 4 hours, and proteins were transferred into a PVDF membrane overnight at 4°C in 1X Tris/Glycine/SDS with 20% methanol. The membrane was blocked in LICOR blocking buffer and hybridized overnight at 4°C with 1:1,300 mouse anti-FMRP (Chemicon, Temecula, CA) and the next day for 2 hours at RT with 1:10,000 anti-mouse secondary antibody (LICOR wv 680 mm). Following detection the blot was re-hybridized with 1: 200,000 mouse anti-GAPDH (Chemicon, Temecula, CA) antibody and the same secondary antibody. Bands were detected at 169 μm resolution using the Odyssey infrared scanner.

#### FMRP immunohistochemistry

Tissue was fixed in paraformaldehyde for at least 48 hours and paraffin embedded. Four μm paraffin-embedded sections of cerebellum and frontal cortex mounted on Thermo Scientific Permafrost Adhesion microscope slides were deparaffinized and hydrated in a series of fifteen minute incubations as follows: Safeclear Xylene substitute (Thermo Fisher Scientific, USA), (v/v) 100% ethyl alcohol (EtOH), 96% EtOH, 70% EtOH and 50% EtOH followed by a five minute incubation in Milli-Q H2O. Slides were washed in 0.1 M Phosphate Buffered Saline (PBS). Heat-mediated antigen retrieval was performed by submerging the slides in Citrate buffer (pH 6; containing 10 mM Citric acid (Sigma) and (v/v) 0.05% Tween-20 (Acros) followed by boiling for 15 minutes in a microwave. Slides were allowed to cool to room temperature (RT) and rinsed in 0.1 M PBS. Endogenous peroxidase activity was quenched by incubating slides in 0.3% H_2_O_2_ for twenty minutes at RT. Slides were rinsed in 0.1 M PBS and incubated for two hours at RT in blocking buffer containing (v/v) 10% fetal donkey serum (Millipore), 0.1% Triton X-100 (Acros), and (w/v) 0.2% gelatin (Acros). Slides were incubated in primary antibody incubation buffer containing mouse anti-FMRP clone 1C3 1:200 (Millipore), (v/v) 2% fetal donkey serum, 0.2% Triton X-100, and (w/v) 0.2% gelatin overnight at RT. Sections were rinsed in 0.1 M PBS and incubated in secondary antibody buffer containing biotin-conjugated donkey anti-mouse secondary antibodies 1:200 (Jackson), (v/v) 2% fetal donkey serum, 0.2% Triton X-100, and (w/v) 0.2% gelatin at RT for one hour. Sections were rinsed in 0.1 M PBS and the immunoenzymatic reaction was visualized with an ABC horseradish peroxidase kit using the chromagen DAB (Vector). Sections were washed in 0.1 M PBS and Milli-Q H2O. Slides were dehydrated in a series of fifteen minute incubations as follows: (v/v) 50% EtOH, 70% EtOH, 96% EtOH, 100% Chloroform, 96% EtOH, 100% EtOH and Safeclear (Fisher) and coverslipped in DPX mounting medium (EMS).

#### Quantification of intranuclear inclusions

Neuropathologic features from single H & E stained 4 μm paraffin sections were quantified from the frontal cortex, hippocampus, temporal cortex, and cerebellum. A priori selection criteria were applied to select anatomically constrained regions of interest and every cell within that region was counted at 1000X total magnification using a Nikon E600 ECLIPSE microscope in the same manner as previously described [26].

## Results

### Clinical history

The patient was a high-functioning man with FXS who was initially diagnosed at age 60 because of behavioral outbursts, which required medical treatment. He was found to be a mosaic for the *FMR1* gene and his IQ at age 60 on the WAIS-R showed a verbal IQ of 69, a performance IQ of 67 and a full scale IQ of 67. His clinical examination demonstrated typical features of FXS including a mildly long face, large and mildly prominent ears, macroorchidism, in addition to perseverative speech, verbal outbursts, paranoid ideation and anxiety that interfered with sleep. He was placed on buspirone and aripiprazole for his behavioral outbursts. At age 71 he developed a history of balance difficulties with falling and slowness in his motor movements. He also described memory problems and his Mini-Mental Status Examination (MMSE) was 25/30. He had masked facies, hypotonic speech and increased tone in the upper extremities without rigidity or tremor. He demonstrated a stooped posture and shuffling gait. Because of his parkinsonian features the aripiprazole was discontinued. His MRI demonstrated subcortical white matter disease and dilated ventricles but he did not have the middle cerebellar peduncle (MCP) sign. Over the next few years he gradually developed more bradykinesia, rigidity and difficulties with walking. He also had depression, which had been an intermittent problem for him since age 60. He was treated with counseling and fluoxetine in addition to his buspirone. At age 77 he had significant cognitive deficits and his MMSE dropped to 13/30. Just before his death he developed an intermittent tremor and he had only limited communication abilities. He was reported in a medical journal [23] and was thought to have Parkinson disease in addition to dementia. He died at age 77 and his brain was extracted for the following neuropathological studies.

The family history was significant in that his brother had the full mutation and fragile X syndrome and was lower functioning cognitively than him. His mother, who was a carrier, died at age 75 and developed lung cancer but had a long history of depression and also had dementia before death.

### Gross and microscopic findings

External examination of the cerebral hemisphere showed moderate cortical atrophy and mild atrophy of the superior and middle lobules of the cerebellar vermis. The lateral ventricle was moderately dilated as seen on serial coronal sections. Numerous representative sections from all regions of the brain were taken. H&E stains were performed on cerebellum, frontal cortex and hippocampus. A severe vascular hyalinopathy was present throughout the brain, and in the cerebrum involving even subcortical vessels. Congophilic angiopathy was not seen.

### *FMR1* molecular measures

**Figure 1:**
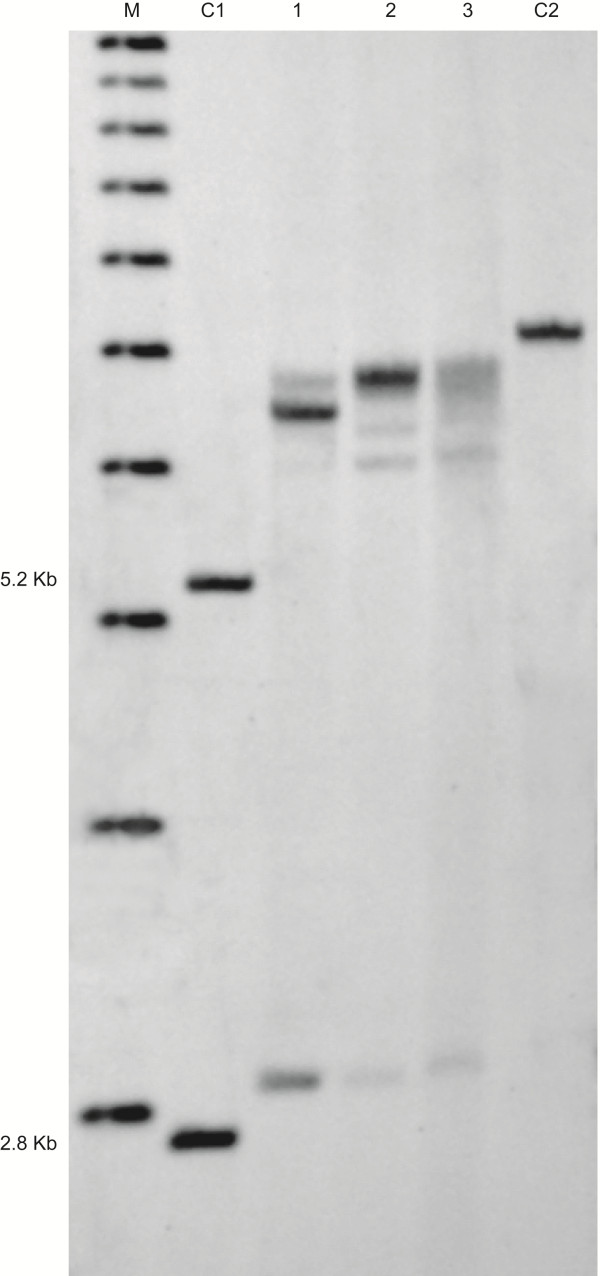
**Southern blot analysis of genomic DNA isolate from two controls: C1 (negative control, normal female with two normal alleles and the corresponding 5.2 Kb and 2.8 Kb bands (methylated and unmethylated alleles respectively) and C2 (positive control, full mutation male with a methylated band of ~ 800 CGG repeats). **Both a premutation allele and a full mutation alleles are present in blood (lane 1), cerebellum (lane 2), and frontal cortex (lane 3) of the proband. DNA marker (M) 1 kb ladder is shown in Lane 1.

*FMR1* mosaicism was identified by Southern Blot and PCR analysis in both peripheral blood and brain tissues. DNA analysis demonstrated the presence of methylated FM alleles (CGG = 300, 440, and 530) and a PM allele in ~25% of cells (78 CGG repeats) in peripheral blood. Genotyping analysis of DNA isolated from post-mortem brain tissues showed that similar *FMR1* mutation patterns (CGG = 300, 390, 530 and 78 in cerebellum, and CGG = 320, 400, 600 and 78 in frontal cortex) were conserved in the brain (Figure 1). However, the proportion of blood cells (percent of methylation) carrying a full mutation was different in peripheral blood (~75%) compared to what observed in both cerebellum (94%) and in frontal cortex (95%) suggestive of greater depletion of the premutation allele in the brain.

**Figure 2:**
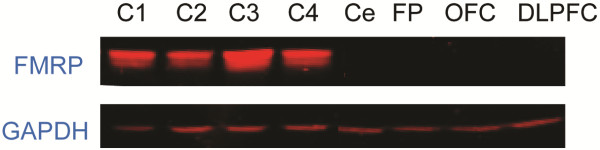
**WB shows lack of FMRP in cerebellar and frontal cortex protein extracts. ** Cerebellar protein extracts from 4 typically age-matched developing controls show the presence of FMRP (75 KDa). No FMRP expression was detected in extracts from cerebellum (Cer) and 3 different frontal cortex regions (frontal pole (FP), orbital frontal cortex (OFC); and dorso lateral pre-frontal cortex (DLPFC). GAPDH (37 KDa) was used as a loading control.

**Figure 3:**
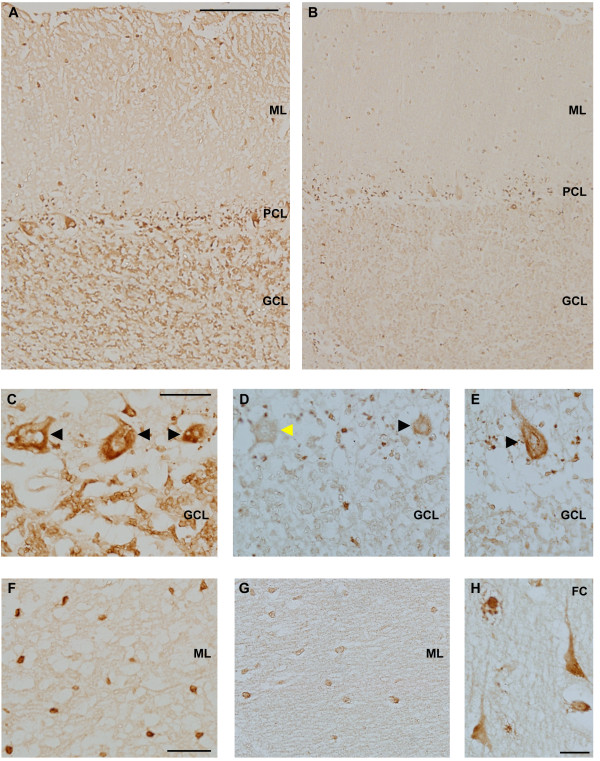
**Immunohistochemistry demonstrates reduced FMRP levels in the cerebellum and frontal cortex of the proband. **Paraffin embedded tissue sections from the proband and controls were immunostained with anti-FMRP (clone 1C3) antibody. (**A,B**): A comparison of FMRP immunoreactivity (FMRP-IR) in the cerebellar cortex of SK relative to age-matched control tissue demonstrates reduced FMRP-IR in the molecular layer (ML), purkinje cell layer (PCL) and granule cell layer (GCL). (**C**) FMRP immunoreactive purkinje cells present in cerebellar cortex of a control subject. The granule cell layer is characterized by significant FMRP expression in control sections which is dramatically reduced in the proband. (**D, E**) FMRP-positive (black arrowhead) and FMRP-negative Purkinje cells (yellow arrowhead) were present in the cerebellum of SK. (**F**) FMRP-positive cells in the molecular layer of cerebellar cortex of a control section. (**G**) Levels of FMRP are significantly reduced in the molecular layer of SK. (**H**) FMRP-positive cells with the morphology of excitatory neurons in the frontal cortex (FC) of SK. Scale bar in A = 200 μm, applies to **A,B**. Scale bar in C = 50 μm, applies to **C-E**. Scale bar in F = 50 μm, applies to **F,G**. Scale bar in H = 25 μm.

*FMR1* mRNA gene expression analysis showed ~ 0.4 fold expression levels from the normal in peripheral blood [23]. Although only ~5-6% of cells carried a premutation allele in brain they were transcriptionally active; indeed the presence of *FMR1* mRNA was detectable in both, the cerebellum (0.06 ± 0.001) and frontal cortex (0.05 ± 0.008). Brain sections from the same brain block that were used for determining CGG repeat size by Southern Blot and *FMR1* mRNA levels by QRT-PCR were used for determining FMRP expression by Western Blot (WB) analysis and immunohistochemistry (IHC). WB analysis did not detect any FMRP (Figure 2). However, we found that despite the dramatic reduction of *FMR1* expression and absence of FMRP signal by WB, IHC showed detectable levels of FMRP expression (Figure 3). Qualitative examination and distribution of FMRP expression in the cerebellum and dorsolateral pre-frontal cortex compared to a normal age-matched control by IHC, which amplifies antigen signal allowing detection of low levels of protein expression, showed numerous cerebellar Purkinje and molecular layer cells immunoreactive for FMRP. FMRP-positive and FMRP-negative cells, in addition to the overall reduction in immunoreactivity, were observed compared to a normal age matched control. FMRP immunoreactive cells with the morphology of excitatory neurons in the dorsolateral pre-frontal cortex were also present (Figure 3). Based on both WB and IHC analyses we qualitatively estimate that the proband presented an approximately ~95% reduction of FMRP expression, consistent with his FXS phenotype. Importantly, similar visualization of sparse, but clearly positive FMRP staining by IHC in the absence of an FMRP-corresponding band on WB has been reported in three cases of FXS [26].

### Quantitative analysis of intranuclear inclusion number

**Figure 4:**
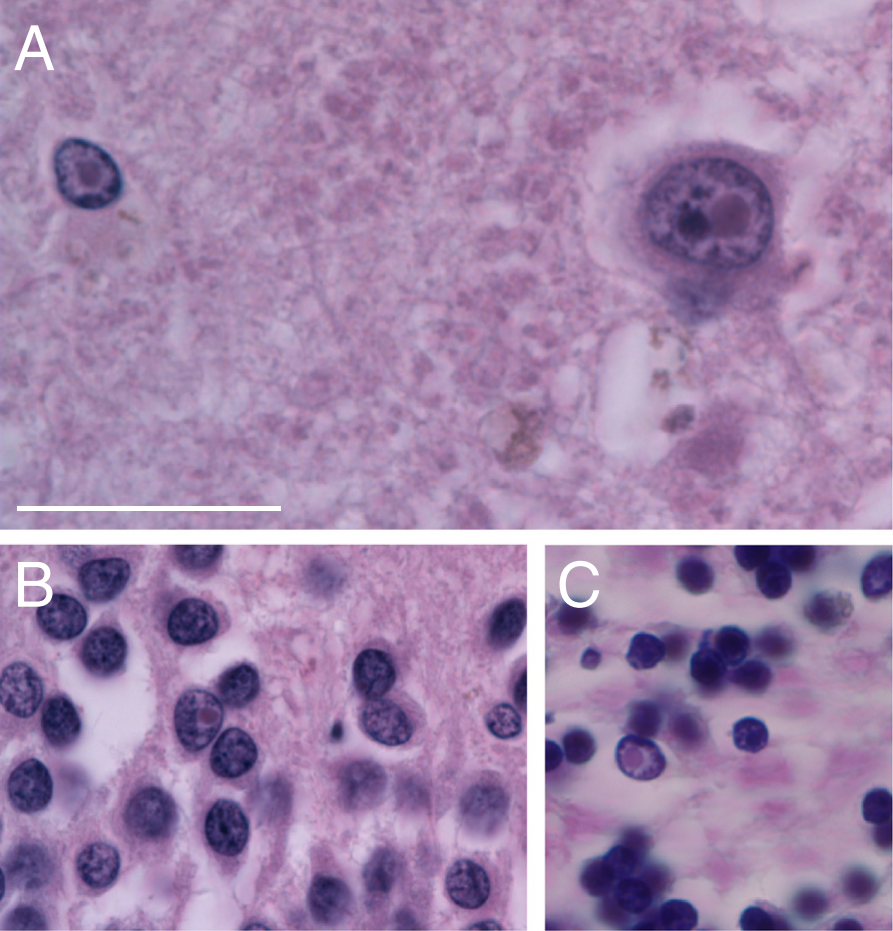
**Intranuclear Inclusions. **(**A**) A pyramidal neuron and an astroglial cell in the frontal cortex containing intranuclear inclusion bodies. Note the pyramidal cell nucleolus adjacent to the intranuclear inclusion and the heterochromatin of the astroglial cell gathered up against the edges of the nucleus. (**B**) Granule neuron in the dentate gyrus of the hippocampus with an intranuclear inclusion body. (**C**) Granule neuron in the cerebellar granule cell layer with an intranuclear inclusion. Note the heterochromatin is pushed away from the inclusion, forming a dense gathering at the nuclear envelope. Scale bar = 50 μm applies to all plates.

To examine the contribution of the PM allele to the patient’s decline during aging we asked whether intranuclear inclusions were present in this individual that would suggest the presence of FXTAS pathology. Throughout this examination we largely focused on the granule cell population as studies in the CGG KI mouse model of PM have shown similar numbers of inclusions in cerebellar, olfactory bulb, and hippocampal granule cell populations [31-33]. Intranuclear inclusions were observed in three different regions of the brain (Figure 4). See Table 1 for a comparison of the present quantifications with those reported previously by [8,26,34].

**Table 1:** Intranuclear inclusion counts

	**Frontal Cortex**		**Temporal Cortex**		**Cerebellum**		**Hippocampus--CA1**		**Hippocampus--DG**	
	**(Gray Matter)**		**(Gray Matter)**		**(Granule Cell Layer)**		**(Pyramidal Cell Layer)**		**(Granule Cell Layer)**	
	**Neurons**	**Astroglia**	**Neurons**	**Astroglia**	**Neurons**	**Astroglia**	**Neurons**	**Astroglia**	**Neurons**	**Astroglia**
Case 1	7 of 206	19 of 421	4 of 189	25 of 311	30 of 1424	42 of 644	5 of 122	11 of 245	15 of 532	8 of 126
mean	**3.40%**	**4.51%**	**2.12%**	**8.03%**	**2.10%**	**6.52%**	**4.09%**	**4.49%**	**2.82%**	**6.35%**
Greco et al., 2006 [8]	4.4%	16.7%	na	na	na	na	10.1%	10.3%	2.1%	26.6%
Tassone et al., 2012 [34]	5.6%	4.24%	11.59%	9.85%	na	na	6.59%	8.84%	4.66%	11.56%
Hunsaker et al., 2011	0.4%	0.93%	na	na	na	na	na	na	0.68%	1.34%

*na *Data not available.

This table shows the number of neurons and astroglia containing intranuclear inclusions in this Case as a function of the number of cells counted. Also included are intranuclear inclusion prevalence data from male FXTAS cases reported by [8], female FXTAS cases reported by [37], and male FXS reported by [26] to provide context for where along the fragile X associated disorder spectrum this size mosaic case fits.

Examination of a coronal hippocampal section from a region proximate to the level of the retrosplenial cortex identified the presence of intranuclear inclusions in low numbers and with irregular distribution. As can be seen in Table 1, when every granule cell and astrocyte in the granule cell layer of the dentate gyrus were counted, the percentage of cells with intranuclear inclusions was estimated to be 2.82% in granule cell nuclei (15 inclusions in 532 cells counted) and 6.35% in astroglial nuclei (8 of 126 cells counted). The granule cell layer was chosen as a region of interest to compare our current estimates with previous accounts which approximated 1% to 7% of granule cells and >25% astrocytes in the granule cell layer containing intranuclear inclusions in male and female cases with FXTAS [8,34]. In addition, hippocampus sections from 5 age-matched, non-FXS control cases reported by [26] were counted using identical methods, and no intranuclear inclusions were observed in any control case.

Morphological analysis allowed clear definition of the CA1/subiculum border and the CA3/CA1 border. Pyramidal neurons and astroglial cells were counted in the pyramidal cell layer in CA1. The percentage of cells with intranuclear inclusions was estimated to be 4.09% of pyramidal neurons (5 of 122 cells counted) and 4.49% of astroglial cells (11 of 245 cells counted). The CA3 and the Hilus were, however, not well represented in these sections and were not counted. Every neuron and astroglial cell was counted in this section similar to the dentate gyrus granule cells.

Similar to the quantifications in the hippocampus, the frontal cortex and temporal cortex were sampled. In this case, a single gyrus was chosen by marking the slide prior to placing under the microscope. The gyrus and sulci on either side were completely sampled and all neurons and astroglia were counted, as well as the number of intranuclear inclusions in each cell type (Temporal cortex: 2.12% of neurons (4 of 189 cells) and 8.03% of astroglial cells (25 of 311 cells); Frontal cortex: 3.40% of neurons (7 of 206 cells) and 4.51% of astroglial cells (19 of 421 cells). The temporal cortex and frontal cortex were chosen for comparison with previous studies of inclusions in male and female subjects with FXTAS [8,34], as well as a report in FXS [26].

To quantify the presence of intranuclear inclusions in the cerebellum, a protocol used to quantify neuropathological features in FXS [35] was modified to quantify inclusions. Prior to placing the section under the microscope, the slide was marked to select a single cerebellar folium. The selection criterion was to select the folium that showed the fewest histological artifacts (i.e., tissue shredding, separation of granule cell layer from Purkinje cell layer, etc.). The internal granule cell layer in a cerebellar section was sampled and all neurons and astroglial quantified. The observed number of intranuclear inclusions in this population was 2.10% of granule cells (30 of 142 counted) and 6.52% of astroglial cells (42 of 642 counted). The Purkinje cell population was also sampled, but no inclusions were identified. The molecular layer was not rigorously sampled, but inclusions were present in very low numbers (i.e., <<1%). A summary of intranuclear inclusion estimates is provided on Table 1.

## Discussion

This study examines the case of a male with *FMR1* size mosaicism consisting of a FM and a PM allele who was diagnosed with FXS and whose phenotype was related to the striking reduction of FMRP expression in the brain. Later in life this individual also showed features congruent with the minor diagnostic criteria for FXTAS, but this diagnosis was not given due to lack of appreciable *FMR1* mRNA levels and the presence of FXS symptomatology [23]. However, upon post mortem analysis we observed the presence of intranuclear inclusions throughout the brain, one of the major diagnostic criteria for FXTAS [27]. *FMR1* mRNA expression, most likely due to the presence of a PM allele in a small percent of cells, was detected in peripheral blood [23] and in two brain regions in this study. Although the overall level of *FMR1* mRNA throughout the brain and in different brain regions (cerebellum and frontal cortex) did not seem elevated, it was likely to be elevated in cells that were actually transcriptionally active (cells with a PM allele) and therefore leading to RNA toxicity due to the presence of the CGG repeat expanded alleles as reported in other cases [26]. Thus, the presence of toxic expanded mRNA is likely responsible for the FXTAS phenotype observed in this individual.

It has been shown that expansion of the CGG repeat leads to sequestration of a number of proteins, including DROSHA and DGCR8, which are key players in the microRNA biogenesis as described in [16] and indeed a dysregulation of the miRNA pattern has been demonstrated in neural cells from FXTAS cases [17]. It is interesting that a small percentage of cells expressing the *FMR1* premutation, as little as 5-6% in the cerebellum and in the frontal cortex, may contribute to a process of neural degeneration in this individual in addition to his parkinsonism. As cells in our brain are involved in many networks, alterations such as protein sequestration and mRNA toxicity in small pockets throughout the brain could potentially lead to significant pathway dysregulation (such as microRNA biogenesis and glutamate signaling pathway) amounting to major signaling network breakdown. In addition, when the subject developed symptoms of FXTAS, he had already experienced a great deficit of FMRP and the consequent related phenotype. This may suggest that early in life the presence of the PM allele, producing small levels of FMRP, could have helped to ameliorating some of the FXS phenotype. In fact, the patient was not diagnosed with FXS until late in life at age 60.

The present finding of symptoms and pathological and molecular evidence of FXTAS in a FXS mosaic male, raises a number of diagnostic issues for FXTAS. The finding also raises concerns since, during antemortem investigations this case received a diagnosis of parkinsonism with cognitive decline based on clinical features, rather than a diagnosis of FXTAS [23]. What this case brings to light is the need for increased vigilance and rigor in the application of diagnostic criteria to carriers of expanded CGG repeats as pertaining to FXTAS, separate from other non-FXTAS movement disorders although the two problems, FXTAS and Parkinson Disease, can occur together.

The diagnosis provided by our group was based on the knowledge of the neurobiology of FXS and FXTAS at the time. Most working models of FXS pathogenesis posit that FXS is the result of dramatically reduced or absent expression of biologically active FMRP (i.e., no FMRP expression due to the expansion of the CGG repeats or deletion or mutation in the coding region of the *FMR1* gene). In contrast, FXTAS is believed to result from a toxic RNA gain of function mechanism resulting in alterations of cellular function [36]. In this case, and based on what was heretofore presumed about the neurobiology of FXS and FXTAS, having >90% reduction in FMRP levels and only ~40% of normal mRNA expression levels was sufficient to suggest FXTAS as a potential diagnosis.

During antemortem investigations, the diagnosis of parkinsonism was made rather than FXTAS-though the potential for a patchy presentation of FXTAS-like symptoms was mentioned as a remote, albeit unlikely possibility at the time. After receiving these diagnoses and following the death of this individual, postmortem brain tissue was available for analysis. Similarly to the three cases previously reported [26], a sparse distribution of intranuclear inclusions was present and a patchy expression of FMRP immunoreactivity was apparent in brain. As can be seen in Table 1, the number of inclusions is intermediate to what has been reported before in FXTAS cases [8,37] and the three FXS cases reported [26]. It is uncertain whether the presence of inclusions always means that a diagnosis of FXTAS is certain and perhaps it should serve as markers of cellular processes associated with the presence of expanded CGG repeat-transcripts rather than to be used as diagnostic tool. This suggests that searching for the presence of intranuclear inclusions in post mortem FXTAS brains is akin to applying NIA-Reagan and Braak criteria to post mortem tissue obtained from probable Alzheimer Disease cases; that is that the diagnosis of FXTAS can be confirmed post mortem using intranuclear inclusions as a quantitative marker.

In conclusion, it is likely that the neuropathological changes of FXTAS were present here and that this patient had FXS and FXTAS, and Parkinson Disease.

## Competing interest

Dr. Randi Hagerman has received funding from Roche, Novartis, Seaside Therapeutics, Forest, Curemark and the National Fragile X Foundation for clinical trials in fragile X syndrome and/or autism. She has also consulted with Novartis regarding treatment in fragile X syndrome. All other authors declare that they have no conflict of interests. This work is dedicated to the memory of Matteo.

## Authors’ contributions

TF designed and coordinated the study, drafted the manuscript and participated in data analysis and interpretation of the results. DP participated in the design and coordination, ran experiments, drafted the manuscript and participated in data analysis and interpretation of the results. HMR ran experiments, drafted the manuscript and participated in data analysis and interpretation of the results. CLC ran experiments, drafted the manuscript and participated in data analysis and interpretation of the results. CMG performed pathological analysis and revision of the manuscript. RJH performed clinical evaluation, participated in drafting and revision of the manuscript and participated to the data analysis and clinical interpretation. SCN revised the manuscript and participated in data analysis and interpretation of the results. DAH performed clinical evaluation and participated in revision of the manuscript. PJH revised the manuscript and participated to the data analysis and interpretation of the results. All authors read and approved the final manuscript.

## References

[B1] Sobesky WE Taylor AK Pennington BF Bennetto L Porter D Riddle J Hagerman RJ Molecular/clinical correlations in females with fragile X Am J Med Genet 1996 64 340 345 10.1002/(SICI)1096-8628(19960809)64:2<340::AID-AJMG21>3.0.CO;2-E 8844077

[B2] Guerreiro MM Camargo EE Kato M Marques-de-Faria AP Ciasca SM Guerreiro CA Netto JR Moura-Ribeiro MV Fragile X syndrome. Clinical, electroencephalographic and neuroimaging characteristics Arq Neuropsiquiatr 1998 56 18 23 9686115 10.1590/s0004-282x1998000100003

[B3] McLennan Y Polussa J Tassone F Hagerman R Fragile x syndrome Curr Genomics 2011 12 216 224 10.2174/138920211795677886 22043169 PMC3137006

[B4] Harris SW Hessl D Goodlin-Jones B Ferranti J Bacalman S Barbato I Tassone F Hagerman PJ Herman H Hagerman RJ Autism profiles of males with fragile X syndrome Am J Ment Retard 2008 113 427 438 10.1352/2008.113:427-438 19127654 PMC2629645

[B5] Rogers SJ Wehner DE Hagerman R The behavioral phenotype in fragile X: symptoms of autism in very young children with fragile X syndrome, idiopathic autism, and other developmental disorders J Dev Behav Pediatr 2001 22 409 417 10.1097/00004703-200112000-00008 11773805

[B6] Hagerman PJ Hagerman RJ Fragile X-associated tremor/ataxia syndrome (FXTAS) Ment Retard Dev Disabil Res Rev 2004 10 25 30 10.1002/mrdd.20005 14994285

[B7] Jacquemont S Hagerman RJ Leehey M Grigsby J Zhang L Brunberg JA Greco C Des Portes V Jardini T Levine R Fragile X premutation tremor/ataxia syndrome: molecular, clinical, and neuroimaging correlates Am J Hum Genet 2003 72 869 878 10.1086/374321 12638084 PMC1180350

[B8] Greco CM Berman RF Martin RM Tassone F Schwartz PH Chang A Trapp BD Iwahashi C Brunberg J Grigsby J Neuropathology of fragile X-associated tremor/ataxia syndrome (FXTAS) Brain 2006 129 243 255 16332642 10.1093/brain/awh683

[B9] Greco CM Hagerman RJ Tassone F Chudley AE Del Bigio MR Jacquemont S Leehey M Hagerman PJ Neuronal intranuclear inclusions in a new cerebellar tremor/ataxia syndrome among fragile X carriers Brain 2002 125 1760 1771 10.1093/brain/awf184 12135967

[B10] Greco CM Soontrapornchai K Wirojanan J Gould JE Hagerman PJ Hagerman RJ Testicular and pituitary inclusion formation in fragile X associated tremor/ataxia syndrome J Urol 2007 177 1434 1437 10.1016/j.juro.2006.11.097 17382748

[B11] Hunsaker MR Greco CM Spath MA Smits AP Navarro CS Tassone F Kros JM Severijnen LA Berry-Kravis EM Berman RF Widespread non-central nervous system organ pathology in fragile X premutation carriers with fragile X-associated tremor/ataxia syndrome and CGG knock-in mice Acta Neuropathol 2011 122 467 479 10.1007/s00401-011-0860-9 21785977 PMC3222079

[B12] Berman RF Willemsen R Mouse models of fragile X-associated tremor ataxia J Investig Med 2009 57 837 841 19574928 10.231/JIM.0b013e3181af59d6 PMC2787904

[B13] Tassone F Iwahashi C Hagerman PJ FMR1 RNA within the intranuclear inclusions of fragile X-associated tremor/ataxia syndrome (FXTAS) RNA Biol 2004 1 103 105 10.4161/rna.1.2.1035 17179750

[B14] Iwahashi CK Yasui DH An HJ Greco CM Tassone F Nannen K Babineau B Lebrilla CB Hagerman RJ Hagerman PJ Protein composition of the intranuclear inclusions of FXTAS Brain 2006 129 256 271 16246864 10.1093/brain/awh650

[B15] Jin P Duan R Qurashi A Qin Y Tian D Rosser TC Liu H Feng Y Warren ST Pur alpha binds to rCGG repeats and modulates repeat-mediated neurodegeneration in a Drosophila model of fragile X tremor/ataxia syndrome Neuron 2007 55 556 564 10.1016/j.neuron.2007.07.020 17698009 PMC1994817

[B16] Sellier C Rau F Liu Y Tassone F Hukema RK Gattoni R Schneider A Richard S Willemsen R Elliott DJ Sam68 sequestration and partial loss of function are associated with splicing alterations in FXTAS patients EMBO J 2010 29 1248 1261 10.1038/emboj.2010.21 20186122 PMC2857464

[B17] Sellier C Freyermuth F Tabet R Tran T He F Ruffenach F Alunni V Moine H Thibault C Page A Sequestration of DROSHA and DGCR8 by expanded CGG-repeats RNA alters microRNA processing in fragile X-associated tremor/ataxia syndrome Cell Reportsin press 10.1016/j.celrep.2013.02.004 PMC3639429 23478018

[B18] Tassone F Hagerman RJ Taylor AK Mills JB Harris SW Gane LW Hagerman PJ Clinical involvement and protein expression in individuals with the FMR1 premutation Am J Med Genet 2000 91 144 152 10.1002/(SICI)1096-8628(20000313)91:2<144::AID-AJMG14>3.0.CO;2-V 10748416

[B19] Kenneson A Zhang F Hagedorn CH Warren ST Reduced FMRP and increased FMR1 transcription is proportionally associated with CGG repeat number in intermediate-length and premutation carriers Hum Mol Genet 2001 10 1449 1454 10.1093/hmg/10.14.1449 11448936

[B20] Primerano B Tassone F Hagerman RJ Hagerman P Amaldi F Bagni C Reduced FMR1 mRNA translation efficiency in fragile X patients with premutations RNA 2002 8 1482 1488 12515381 PMC1370354

[B21] Peprah E He W Allen E Oliver T Boyne A Sherman SL Examination of FMR1 transcript and protein levels among 74 premutation carriers J Hum Genet 2010 55 66 68 10.1038/jhg.2009.121 19927162 PMC4122982

[B22] Hagerman RJ Leehey M Heinrichs W Tassone F Wilson R Hills J Grigsby J Gage B Hagerman PJ Intention tremor, parkinsonism, and generalized brain atrophy in male carriers of fragile X Neurology 2001 57 127 130 10.1212/WNL.57.1.127 11445641

[B23] Hall D Pickler L Riley K Tassone F Hagerman R Parkinsonism and cognitive decline in a fragile X mosaic male Mov Disord 2010 25 1523 1524 10.1002/mds.23150 20568092 PMC4051493

[B24] Liu Y Winarni T Zhang L Tassone F Hagerman R Fragile X-associated tremor/ataxia syndrome (FXTAS) in grey zone carriers Clin Genet 2012 1 4 23009394 10.1111/cge.12026 PMC4991824

[B25] Loesch DZ Sherwell S Kinsella G Tassone F Taylor A Amor D Sung S Evans A Fragile X-associated tremor/ataxia phenotype in a male carrier of unmethylated full mutation in the FMR1 gene Clin Genet 2012 82 88 92 10.1111/j.1399-0004.2011.01675.x 21476992

[B26] Hunsaker MR Greco CM Tassone F Berman RF Willemsen R Hagerman RJ Hagerman PJ Rare intranuclear inclusions in the brains of 3 older adult males with fragile x syndrome: implications for the spectrum of fragile x-associated disorders J Neuropathol Exp Neurol 2011 70 462 469 10.1097/NEN.0b013e31821d3194 21572337 PMC3109086

[B27] Hagerman PJ Hagerman RJ The fragile-X premutation: a maturing perspective Am J Hum Genet 2004 74 805 816 10.1086/386296 15052536 PMC1181976

[B28] Tassone F Pan R Amiri K Taylor AK Hagerman PJ A rapid polymerase chain reaction-based screening method for identification of all expanded alleles of the fragile X (FMR1) gene in newborn and high-risk populations J Mol Diagn 2008 10 43 49 10.2353/jmoldx.2008.070073 18165273 PMC2175542

[B29] Filipovic-Sadic S Sah S Chen L Krosting J Sekinger E Zhang W Hagerman PJ Stenzel TT Hadd AG Latham GJ Tassone F A novel FMR1 PCR method for the routine detection of low abundance expanded alleles and full mutations in fragile X syndrome Clin Chem 2010 56 399 408 20056738 10.1373/clinchem.2009.136101 PMC4031651

[B30] Tassone F Hagerman RJ Taylor AK Gane LW Godfrey TE Hagerman PJ Elevated levels of FMR1 mRNA in carrier males: a new mechanism of involvement in the fragile-X syndrome Am J Hum Genet 2000 66 6 15 10.1086/302720 10631132 PMC1288349

[B31] Hunsaker MR Wenzel HJ Willemsen R Berman RF Progressive spatial processing deficits in a mouse model of the fragile X premutation Behav Neurosci 2009 123 1315 1324 20001115 10.1037/a0017616 PMC3410547

[B32] Schluter EW Hunsaker MR Greco CM Willemsen R Berman RF Distribution and frequency of intranuclear inclusions in female CGG KI mice modeling the fragile X premutation Brain Res 2012 1472 124 137 22796595 10.1016/j.brainres.2012.06.052 PMC3572858

[B33] Wenzel HJ Hunsaker MR Greco CM Willemsen R Berman RF Ubiquitin-positive intranuclear inclusions in neuronal and glial cells in a mouse model of the fragile X premutation Brain Res 2010 1318 155 166 20051238 10.1016/j.brainres.2009.12.077 PMC3086812

[B34] Tassone F Greco CM Hunsaker MR Seritan AL Berman RF Gane LW Jacquemont S Basuta K Jin LW Hagerman PJ Hagerman RJ Neuropathological, clinical and molecular pathology in female fragile X premutation carriers with and without FXTAS Genes Brain Behav 2012 11 577 585 10.1111/j.1601-183X.2012.00779.x 22463693 PMC3965773

[B35] Greco CM Navarro CS Hunsaker MR Maezawa I Shuler JF Tassone F Delany M Au JW Berman RF Jin LW Neuropathologic features in the hippocampus and cerebellum of three older men with fragile X syndrome Mol Autism 2011 2 2 14 10.1186/2040-2392-2-2 21303513 PMC3045897

[B36] Hagerman PJ Current gaps in understanding the molecular basis of FXTAS Tremor Other Hyperkinet Mov (N Y) 2012 2 1 7 10.7916/D80C4TH0 PMC3379894 23440729

[B37] Tassone F Hagerman R The fragile X-associated tremor ataxia syndrome Results Probl Cell Differ 2012 54 337 357 10.1007/978-3-642-21649-7_18 22009361

